# Mitigation of Imidacloprid Toxicity in Poultry Chicken by Selenium Nanoparticles: Growth Performance, Lipid Peroxidation, and Blood Traits

**DOI:** 10.1007/s12011-023-03592-5

**Published:** 2023-02-15

**Authors:** Yahya Z. Eid, Yassin Omara, Asmaa Ragab, Ahmed Ismail, Mohsen Zommara, Mahmoud A. O. Dawood

**Affiliations:** 1 Department of Poultry Science, Faculty of Agriculture, Kafrelsheikh University, Kafr El-Sheikh, 33516 Egypt; 2 Department of Pesticides, Chemistry and Toxicology, Faculty of Agriculture, Kafrelsheikh University, Kafrelsheikh, Egypt; 3 Department of Dairy Production, Faculty of Agriculture, Kafrelsheikh University, Kafrelsheikh, 33516 Egypt; 4 Animal Production Department, Faculty of Agriculture, Kafrelsheikh University, Kafr El-Sheikh, Egypt; 5 The Center for Applied Research on the Environment and Sustainability, The American University in Cairo, Cairo, 11835 Egypt

**Keywords:** Trace minerals, Nanotechology, Insecticdes, Poultry, Blood biomarkers

## Abstract

Imidacloprid is an insecticide that protects against insects in the agriculture, animal, and poultry production sectors. Since the accumulation of imidacloprid induces adverse impacts on general health status and quality of the food chain, this study tested the impacts on broilers. Besides, selenium nanoparticles were fed to birds to relieve the negative impacts on growth performance and health status. Birds (1-day age, initial weight 46.05 ± 1.0 g) divided into four groups (triplicates) where 15 chicks of each replicate (45 for each group). The first group (control) was fed the basal diet without either selenium or imidacloprid toxicity. The second group was fed selenium nano form at 3 mg/kg. The third group was fed selenium and exposed to imidacloprid at 1/10 LT_50_ (3 mg/kg body weight). The fourth group was fed selenium nano form (3 mg/kg) and exposed to imidacloprid at 1/10 LT_50_ (3 mg/kg body weight). All groups were kept under the same conditions for 35 days. The final weight and weight gain of birds fed selenium nano form showed marked improvement compared to the imidacloprid-exposed group, while the feed intake and feed conversion ratio markedly reduced. The red blood cells showed higher values in birds fed selenium nano than the control and those exposed to imidacloprid. Interestingly, the hemoglobulin and hematocrit increased in birds fed selenium nano form with or without imidacloprid exposure. Furthermore, the white blood cells increased in birds fed selenium nano form with or without imidacloprid exposure. The total protein, albumin, and globulin were higher in birds fed selenium nanoparticles than those exposed to imidacloprid with or without selenium feeding. Birds in the control and imidacloprid groups had higher aspartate aminotransferase (AST), alanine aminotransferase (ALT), and malondialdehyde levels than the remaining groups. Accordingly, dietary selenium nanoparticles are suggested in broiler feed to cope with the adverse effects of imidacloprid toxicity.

## Introduction

Imidacloprid is a water-soluble insecticide involved in several applications in the agriculture and veterinary sectors [1]. Imidacloprid is one of the neonicotinoids used to combat insects attacking crops and poultry farms [2]. In poultry farms, the high presence of insects requires imidacloprid spray by painting walls and washing utensils [3]. Although imidacloprid is selectively attacking insects and is apparently safe for birds, the lack of knowledge of accurate dose and the possibility of ingestion through water and feed are risky and toxic for birds [4, 5]. Imidacloprid toxicity causes high production of free radicals involved in lipid peroxidation and oxidative stress [6]. Continuous exposure to imidacloprid induces inflammation, immunosuppression, and disruption of antioxidative status in birds [7]. Consequently, birds revealed irregular liver-related biomarkers secretions (alkaline phosphate and transaminase), lipids (cholesterol and triglycerides), and hematological indices [8]. In this context, Emam et al. [3], Gul et al. [9], and Ravikanth et al. [10] reported that including vitamin E, selenium, and silymarin in the food of Japanese quails, chickens, and broilers alleviated the toxic impacts of imidacloprid exposure. Hence, natural antioxidants are suggested to repair the antioxidative capacity, reduce lipid peroxidation, and regulate the birds’ physiological and metabolic functions [6, 8].

As a natural biocursour for selenoproteins formation, selenium is known for its active antioxidation capacity [11, 12]. Besides, selenium has several biological effects, such as antimicrobial, metabolic mediator, and immune enhancer [13]. The absence of selenium induces low production of selenoproteins in birds’ livers leading to high oxidative stress and physiological dysfunction [14]. Recently, biologically synthesized nano selenium applications in the poultry industry revealed several potential roles [15]. In this regard, feeding birds with nano selenium particles caused promotion in the growth performance, enhancement of feed digestion, and regulated blood bioindicators [16, 17]. Furthermore, nano selenium supplementation activated birds’ immunity and antibacterial capacity [18, 19]. Activated antioxidation status was noted in birds treated with nano selenium form [20, 21]. Biologically formed nano Se particles are known for their active surfaces compared with physical and chemical nanoparticles associated with effective nutritional and biological impacts [22]. Beneficial bacterial and yeast strains are normally used to biologically synthesize selenium nanoparticles [23]. Lactic acid bacteria (LAB) recently used to produce highly effective selenium nanoparticles [24]. The produced selenium nanoparticles were included in bird and fish feeds and resulted in improved growth performance and health status [25]. In broiler feeding, supplementing selenium nanoparticles improved productivity, carcass quality, and health status [9, 15].

Since broiler chickens are the largest animal protein source and are markedly farmed using conventional methods, especially in developing countries, exposure to imidacloprid through water or food is expected [26]. The current study proposed that biologically formed selenium nanoparticles can mitigate the negative impacts of imidacloprid toxicity on broilers. Furthermore, this study evaluated lipid peroxidation induced by imidacloprid toxicity on growth performance, blood traits, and feed digestion.

## Materials and Methods

### Ethics Approval

The ethics review board of the Institutional Animal Care and Use Committee at Kafrelsheikh University, Kafrelsheikh, Egypt, approved the experimental procedure.

### Imidacloprid

Imidacloprid (Confidor 17.8% SL) was obtained from Bayer Crop Science Limited, and LD_50_ of malathion was determined according to Finney [27]. The treated dose calculated to be equivalent to 1/10 of the determined LD_50_ by following Kammon et al. [28] and Emam et al. [3]. Daily feeding intubation of imidacloprid was extended for 4 weeks, while the control group was intubated with distilled water and kept under the same conditions.

### Selenium Nanoparticles

Biologically synthesized selenium nano form using *Lactobacillus delbrueckii* subsp. bulgaricus (NCAIM B 02,206) and *Streptococcus thermophilus* (CNCM I-1670) were prepared by following Prokisch et al. [29] and Dawood et al. [30]. The cell pellets were washed two times with Tris–HCl buffer (50 mM, pH 7.5), and finally with ultra-pure water to obtain the Se nanoparticle-fortified cell fraction. Selenium determination carried out according to the method previously described by Zommara et al. [31].

Scanning electron microscope (SEM) (JSM-IT100, JEOL Co. Japan) photos of pure selenium nanoparticles were used for selenium nanoparticles size determination, according to Nagy et al. [32].

### Birds and Experimental Procedure

The study was done at the Poultry Experimental Station, Faculty of Agriculture, Kafr El-Sheikh University, Kafr El-Sheikh Governorate, Egypt. All chicks were brooded together on the first day; 15 chicks of each replicate (45 for each group), having average body weight around the d-old chick weight (46.05 ± 1.0 g), were used to measure their growth performance up to 35 days of age. Birds were allowed free access to feed and water during the fattening period. The birds kept in 63-floor pens (3 m^2^), which covered with wood shavings as litter material. Each pen was equipped with two hanging feeders and one drinker. The lighting cycle was 24 h/day maintained. Chicks were fed by basal diet 21% CP and 3100 kcal ME/kg (Table 1). The chicks received a fixed diet until 1 to 35 days of age. The experimental diets were formulated to cover the nutrient requirement of broiler chicks from 1 to 35 days according to Cobb-Vantress [33] and experimental conditions. Birds were allocated into four groups, where the first group (control) was fed the basal diet without either selenium or imidacloprid toxicity. The second group was fed the basal diet with selenium nano form at 3 mg/kg. Selenium nano form particles were added to the diets at 3 mg/kg following Ibrahim et al. [25] and Gul et al. [9] without imidacloprid toxicity. The third group was fed the basal diet without selenium and exposed to imidacloprid at 1/10 LT_50_ (3 mg/kg body weight). The fourth group was fed a basal diet with selenium nano form (3 mg/kg) and was exposed to imidacloprid at 1/10 LT_50_ (3 mg/kg body weight). All groups were kept under the same conditions for 35 days.

**Table 1:** Ingredients and chemical composition of the basal diet

Ingredients	%	Chemical analysis	
Yellow corn (8.5%)	57.5	Crude protein (%)	21.1
Soybean meal (44%)	31.3	Ether extract (%)	2.89
Corn gluten meal (60%)	2.94	Calcium (%)	1.1
DL-methionine	0.24	Total phosphorus (%)	0.73
Lysine-Hcl	0.18	Metabolizable energy (Kcal/kg)**	3093
Soybean oil	3.66		
Di-calcium phosphate (CaHPO_4_)	1.64		
Pre-mix*	0.3		
Choline chloride	0.1		
Calcium carbonate (CaCo_3_)	1.66		
Sodium chloride (NaCl)	0.35		
Sodium bicarbonate	0.08		
Anti-coccidiosis drug	0.05		
Total	100		

^*^Pre-mix each 3 kg of vitamin and mixture contains: 13,000,000 IU vit. A; 5,000,000 IU D3 8000 mg E; 4000 mg K; 5000 mg B1; 9000 mg B2; 4000 mg B6; 20 mg B12; 15,000 mg pantothenic acid; 6000 mg nicotinic acid; 2000 mg folic acid; 150 mg biotin; 40,000 mg choline chlorine; 20,000 mg copper sulfate; 1000 mg Ca iodide; 50,000 mg ferrous sulfate; 10,000 mg manganese oxide; 100,000 mg zinc oxide; and 300 mg sodium selenite

^**^Values were calculated according to NRC [34]

### Growth Efficiency

The amount of feed intake was recorded during the trial. Live body weight, body weight gain (WG), and feed conversion ratio (FCR) were calculated at the end of the trial.

$$\begin{array}{l}\mathrm{WG}\left(\mathrm g\right)=\mathrm{Final}\;\mathrm{body}\;\mathrm{weight}\;\left(\mathrm g\right)-\mathrm{Initial}\;\mathrm{body}\;\mathrm{weight}\;\left(\mathrm g\right)\\\mathrm{FCR}=\mathrm{Feed}\;\mathrm{intake}\left(\mathrm g\right)/\mathrm{WG}\left(\mathrm g\right)\end{array}$$

### Blood Sampling and Analysis

Then six birds per replicate were randomly chosen and slaughtered to collect blood. Heparinized whole blood was analyzed after collection by 2 h for estimation of red blood cells (RBCs) count, packed cell volume (PCV), hemoglobin concentration, mean corpuscular volume (MCV), mean corpuscular hemoglobin (MCH), mean corpuscular hemoglobin concentration (MCHC), white blood cells (WBCs) according to Jain [35]. Then, blood smears were prepared from each blood sample on two clean microscope slides. Slides were left to dry at room temperature. Slides were stained with a modified Wright’s stain and covered. One hundred cells were counted under × 100 lense, and the number of neutrophils count, lymphocyte count, monocyte, eosinophil, and basophil were calculated. Five milliliters of blood samples were collected and centrifuged at 3000 rpm for 20 min. Plasma was then collected and kept at − 20 °C for biochemical analysis. Blood parameters, including total protein, albumin, aspartate aminotransferase (AST), and alanine aminotransferase (ALT), were assessed calorimetrically using commercial kits (Diamond Diagnostics, Egypt) following the manufacturer outlines. Malondialdehyde (MDA) in plasma was determined enzymatically using kits from Biodiagonstic, Dokki, Giza, Egypt.

### Statistical Analysis

One-way ANOVA analyzed the collected data in a completely randomized design using IBM SPSS Statistics version 26 (IBM Corp. Released 2019. IBM SPSS Statistics for Windows, Version 26 Armonk, NY: IBM Corp). The significance of means’ differences was tested using the Tukey test, and all differences were considered significant at *P* ≤ 0.05.

## Results

### Selenium Nanoparticles Characterization

**Fig. 1:**
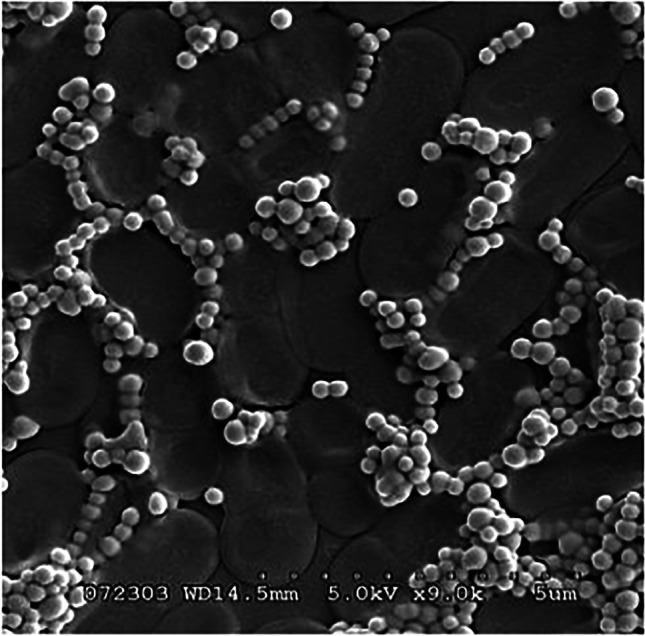
Scanning electron microscope (SEM) photograph of a yogurt culture–selenium nanoparticles suspension

**Fig. 2:**
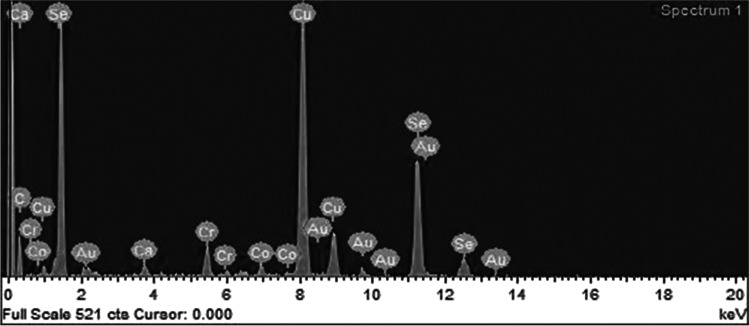
Energy dispersive X-ray spectra of the produced metal spheres

Figure 1 indicates that selenium showed a nanospheres form with a circular shape. Selenium nanoparticles’ size ranged from 55–238 nm with an average of 122.6 ± 34.6 (SD) or 122.6 ± 8.6 (SE) (Fig. 2).

### Growth Performance

The final weight (FBW) and weight gain (WG) of birds fed selenium nano form showed marked improvement compared to the imidacloprid-exposed group (Table 2). However, the feed intake and feed conversion ratio (FCR) were markedly reduced in birds fed selenium nanospheres compared to those exposed to imidacloprid with selenium feeding (Table 2). In addition, no meaningful differences were seen in birds fed selenium nano form and exposed to imidacloprid and the control and birds fed selenium nano form and exposed to imidacloprid in terms of the FBW, WG, feed intake, and FCR.

**Table 2:** The growth performance and feed intake of birds fed with or without selenium (Se) nano form and exposed to imidacloprid for 35 days

	Control	Nano Se	Imidacloprid	Imidacloprid + Nano Se
Final body weight (g)	1904.05 ± 25.26ab	2031.25 ± 27.37a	1842.95 ± 19.58b	1890.85 ± 30.67ab
Weight gain (g)	1950.1 ± 31.7ab	2077.3 ± 44.4a	1889.0 ± 72.7b	1936.9 ± 38.9ab
Feed intake (g)	3190.36 ± 45.69ab	3072.33 ± 52.72b	3230.19 ± 64.38a	3114.54 ± 57.39ab
Feed conversion ratio	1.64 ± 0.01ab	1.48 ± 0.02b	1.71 ± 0.05a	1.61 ± 0.01ab

Values ± SE within a row with different superscripts are significantly different (*P* ≤ 0.05) (*n* = 3)

### Blood Hematology

The red blood cells showed higher values in birds fed selenium nano than the control and those exposed to imidacloprid, while no difference was seen with that fed selenium and exposed to imidacloprid (Table 3). Interestingly, the hemoglobulin and hematocrit were higher in birds fed selenium nano form with or without imidacloprid exposure than the control and those exposed to imidacloprid (Table 3). Furthermore, the white blood cells (WBCs) were higher in birds fed selenium nano form with or without imidacloprid exposure than the control and those exposed to imidacloprid (Table 3). Birds in the control had higher WBCs than those exposed to imidacloprid without selenium feeding.

**Table 3:** The hematological indices of birds fed with or without selenium (Se) nano form and exposed to imidacloprid for 35 days

	Control	Nano Se	Imidacloprid	Imidacloprid + Nano Se
RBCs	2.34 ± 0.1b	2.56 ± 0.1a	2.29 ± 0.1b	2.48 ± 0.07ab
Hb	11.8 ± 0.3b	12.4 ± 0.5a	11.3 ± 0.4b	12.1 ± 0.4a
Hct	24.91 ± 0.8b	26.65 ± 2.1a	23.30 ± 0.1b	26.24 ± 0.6a
MCV	106.7 ± 3.8	104.33 ± 1.8	101.57 ± 4.3	105.81 ± 0.6
MCH	50.43 ± 0.8	48.44 ± 1.9	49.34 ± 0.8	48.79 ± 0.5
MCHC	47.43 ± 1.2	46.53 ± 1.6	48.60 ± 1.2	46.11 ± 0.4
WBCs	136.0 ± 2.9b	147.5 ± 3.5a	129.6 ± 3.4c	143.5 ± 3.0a
LYM %	82.00 ± 2.1	86.00 ± 0.6	82.00 ± 0.6	83.00 ± 0.3
Monocytes	7 ± 1.6	6.4 ± 0.3	7 ± 1.0	6 ± 0.4
Eosinophil	1 ± 0.5	2 ± 0.5	1 ± 0.4	1 ± 0.1
Basophil	1 ± 0.3	2 ± 0.4	1 ± 0.2	1 ± 0.2

Values ± SE within a row with different superscripts are significantly different (*P* ≤ 0.05) (*n* = 3). Red blood cells (RBC), hemoglobin concentration (Hb), hematocrit (Hct), mean corpuscular volume (MCV), mean corpuscular hemoglobin (MCH), mean corpuscular ha hemoglobin concentration (MCHC), white blood cells (WBCs), and lymphocytes (LYM)

### Blood Protein Profile

**Fig. 3:**
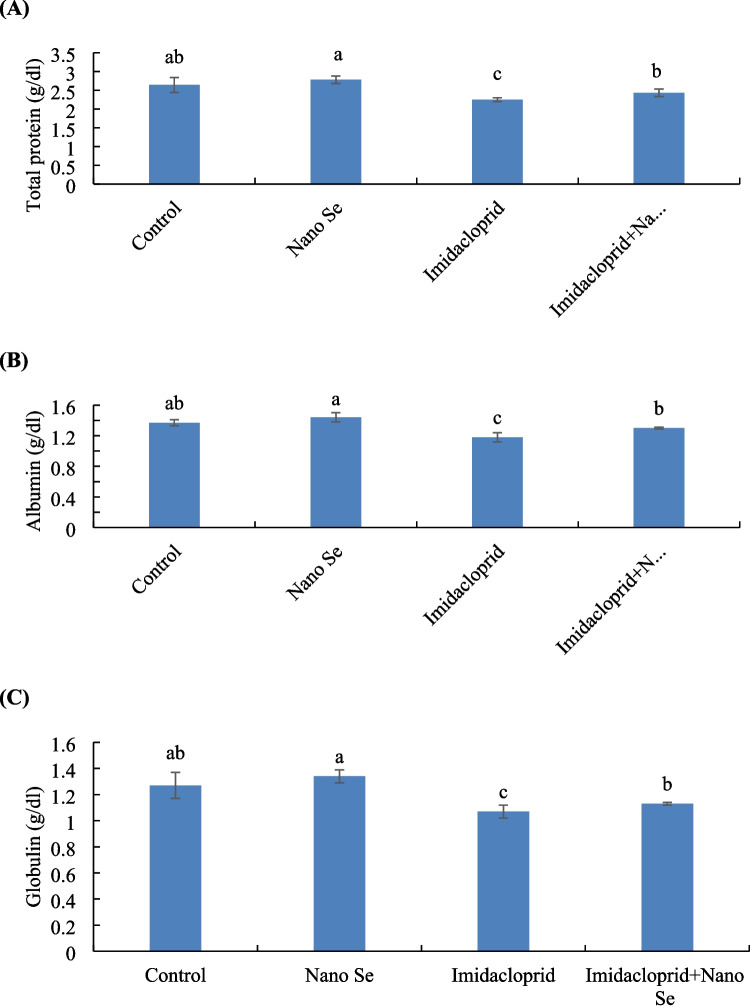
The blood protein profile: (**A**) total protein, (**B**) albumin, and (**C**) globulin of birds fed with or without selenium (Se) nano form and exposed to imidacloprid for 35 days. Values ± SE with different superscripts are significantly different (*P* ≤ 0.05) (*n* = 3)

The total protein, albumin, and globulin were higher in birds fed selenium nanoparticles than those exposed to imidacloprid with or without selenium feeding (Fig. 3). At the same time, no significant difference was detected between the control and selenium-fed group. Markedly, birds exposed to imidacloprid and fed selenium nano form had higher total protein, albumin, and globulin values than those exposed to imidacloprid without selenium feeding.

### Liver Biomarkers

**Fig. 4:**
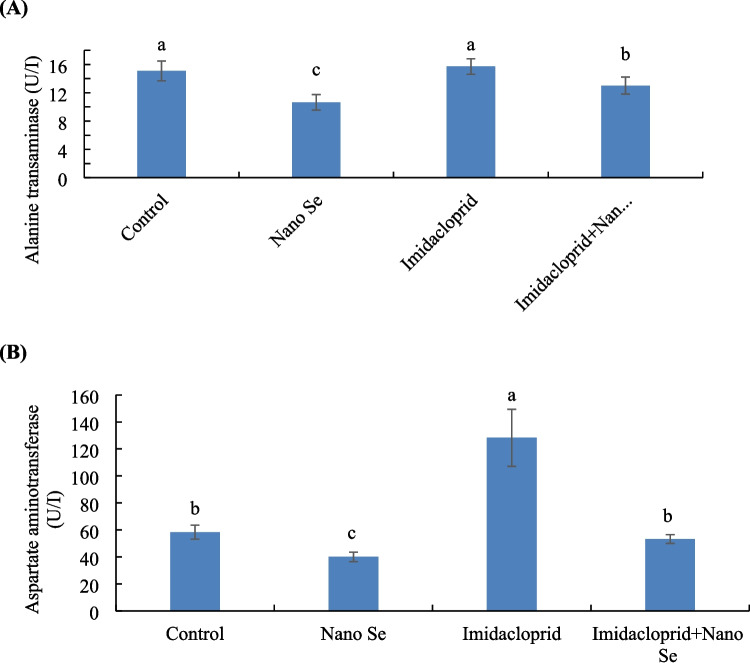
Liver biomarkers: (**A**) alanine transaminase and (**B**) aspartate aminotransferase of birds fed with or without selenium (Se) nano form and exposed to imidacloprid for 35 days. Values ± SE with different superscripts are significantly different (*P* ≤ 0.05) (*n* = 3)

Birds in the control and imidacloprid groups had higher aspartate aminotransferase (AST) than the remaining groups, while birds fed selenium nano form had lower AST than the remaining groups (Fig. 4(A)). Furthermore, birds fed selenium and exposed to imidacloprid had lower AST than birds exposed to imidacloprid without selenium feeding.

Birds in the imidacloprid group had higher alanine aminotransferase (ALT) than the remaining groups, while birds fed selenium nano form had lower ALT than the remaining groups (Fig. 4(B)). Furthermore, for birds fed selenium and exposed to imidacloprid, the control group had lower ALT than birds exposed to imidacloprid without selenium feeding.

### Lipid Peroxidation

**Fig. 5:**
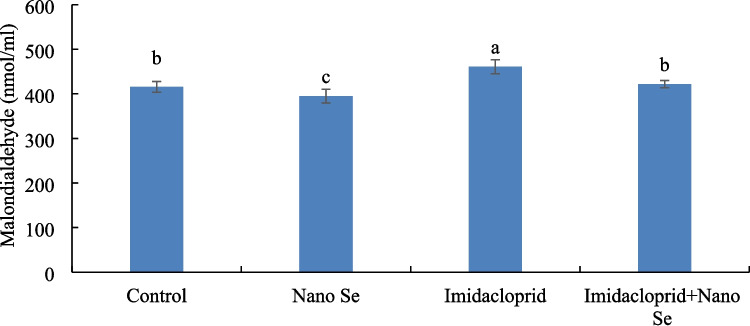
Lipid peroxidation (malondialdehyde level) of birds fed with or without selenium (Se) nano form and exposed to imidacloprid for 35 days. Values ± SE with different superscripts are significantly different (*P* ≤ 0.05) (*n* = 3)

Birds in the imidacloprid group had higher malondialdehyde (MDA) levels than the remaining groups, while birds fed selenium nano form had lower MDA than the remaining groups (Fig. 5). Furthermore, for birds fed selenium and exposed to imidacloprid, the control group had lower MDA than birds exposed to imidacloprid without selenium feeding.

## Discussion

Insecticides are globally applied to enhance crop productivity and veterinary purposes [36]. Subsequently, insecticide derivatives can reach the ecosystem and spoil the food chain supply [5]. Chickens are a valued source of animal proteins, and optimizing their farming conditions is mandatory for saving productivity and ensuring high-quality food for humanity [37]. Selenium nano form is recognized as a functional supplement associated with high antioxidative capacity and vital biological roles in birds [15]. Imidacloprid caused lower feed utilization and growth performance in birds [7], while nano selenium mediated the growth performance [3, 9]. Incorporating nano selenium alleviated the adverse effects of imidacloprid on productive performance. The reduction in the growth performance due to imidacloprid toxicity is in harmony with Ravikanth et al. [10]. Besides, Gul et al. [9] reported a marked reduction in the body weight of birds stressed with imidacloprid. The poor growth performances induced by imidacloprid can be attributed to the adverse impact of imidacloprid on health status [5]. Imidacloprid toxicity disrupts the metabolic function resulting from oxidative stress [38, 39]. Indeed, the overproduction of free radicals (ROS) results from imidacloprid exposure and, thereby, high lipid peroxidation and inflammatory features [40]. Imidacloprid probably reduced the growth performance of birds by affecting feed digestion and absorption in birds’ guts [5]. Besides, the weakened health status resulting from imidacloprid toxicity causes irregular metabolic and physiological responses that coincide with a low growth rate. Markedly, the treatment with selenium nano form recorded significantly higher growth performance than the control and imidacloprid-exposed birds. In the same line, Ibrahim et al. [25] found that selenium nano form addition significantly raised feed intake and improved feed conversion ratio. Further, Khajeh Bami et al. [18] found that adding selenium nano form improved the performance of chickens. Similarly, these results agree with Emam et al. [3], who explained the potent role of selenium nano form in enhancing the productive performance in birds exposed to imidacloprid. Rehman et al. [41] showed the role of selenium nano form in enhancing birds' performance. They attributed these effects to antioxidant activity that stimulated protein synthesis by the bird's enzymatic system and, therefore, the general physiological response. In addition, the results showed improved feed utilization (e.g., feed intake) and decreased FCR in birds treated with selenium nano form. The enhanced feed utilization can explain the improvement in broilers’ growth performance, which results from the roles of selenium in increasing intestinal health and digestion capacity by increasing the villi length [18].

Farmed chickens are vital sources of high-quality animal protein. Therefore, food safety standards must be confirmed to ensure the validity of birds needed for humanity [37]. By detecting hematological indices, birds’ performances can be detected in a low-cost way for health status assessment [42]. Red blood cells (RBCs) are involved in oxygen transportation to the entire body tissues, and lacking RBCs induces failure physiological and metabolic impacts [43]. Hemoglobin (Hb) also participates in oxygen transportation, providing the energy needed for cellular metabolic function. In addition, the white blood cells (WBCs) or leukocytes are part of the immune system and defend against infection through different responses [44]. Hematocrit value (Hct) implies the efficiency of nutrition and availability of nutrients, whereas a low level of Hct indicates anemic features [45]. This study evaluated the hematological profile of birds exposed to imidacloprid with or without selenium nano form feeding. The results showed a reduction in RBCs, Hb, Hct, and WBCs of birds exposed to imidacloprid while selenium nano form mediated the values of hematological indices. Similarly, Gul et al. [9] reported that chickens exposed to imidacloprid had impaired RBCs, Hb, Hct, and WBCs. The reduction of RBCs, Hb, Hct, and WBCs in birds can be related to oxidative stress, impaired immunity, and metabolism failure in birds exposed to imidacloprid [38, 39].

Detecting blood biomarkers is essential for evaluating birds’ health conditions, especially when stressed with biotic or abiotic stressors [42]. Physiologically, stressed birds by insecticides reveal interruption in feed digestion, metabolism, and thereby irregular blood values [36]. This study evaluated blood proteins and liver function-related biomarkers in birds exposed to imidacloprid with or without selenium nano form feeding. The reduction in total protein, albumin, and globulin values in the blood samples of birds exposed to imidacloprid could be due to the inhibition of hepatic protein synthesis at the post-transcription stage by competitive inhibition of phenylalanine-t-RNA synthesis, thus stopping amino-acylation and peptide elongation [46]. One of the consequential impacts of imidacloprid is reducing the absorption of digested amino acids from birds’ gut to the blood downstream, which reduces the blood protein profile [4, 5]. However, the treatment with selenium nano form mitigated the adverse effects of imidacloprid on the blood protein profile that is probably associated with the role of selenium as a metabolic regulator. Indeed, selenium could regulate the absorption of nutrients in birds’ guts and enhance feed utilization so affordable nutrients can be available in the blood for metabolic functions [47]. Furthermore, selenium stimulates hepatic cells and induces ribosomal RNA synthesis to promote protein production [48].

The hepatic tissue mainly removes and detoxifies xenobiotics that harm birds [49]. The liver-related biomarkers (ALT and AST) showed higher levels in imidacloprid-exposed birds increased significantly than the control and selenium-treated birds. The elevation in ALT and AST levels could be attributed to enzyme leakage due to liver damage [3]. Our findings agree with Emam et al. [3], who found that imidacloprid-contaminated birds showed increased ALT and AST. Imidacloprid toxicity has hepatotoxic effects associated with the high release of liver-related enzymes (ALT and AST) [38, 39]. In the case of imidacloprid exposure, lipid peroxidation may be the reason for the uncontrolled secretion of ALT and AST. On the other hand, selenium nano form reduced the ALT and AST levels in birds exposed or not exposed to imidacloprid. Similarly, selenium supplementation reduced the levels of ALT and AST in birds. The reduction of liver biomarkers may be due to the potent effect of selenium and its mechanism of action, mainly as an antioxidant resource that can reduce oxidative stress and protect hepatic cellular function [15].

The toxicity of pesticides induces the overproduction of ROS involved in oxidative stress and, thereby, high malondialdehyde (MDA) levels [7]. When the levels of MDA exceed the antioxidative capacity, ROS impairs the cellular DNA and causes severe cellular damage [50, 51]. Hence, the disruption in the metabolism, physiological, immunological, and growth rate of birds exposed to imidacloprid toxicity can be related to the high MDA levels. The results showed high MDA levels in birds exposed to imidacloprid, while selenium nano form reduced the MDA level. Concisely, Emam et al. [3] and Abd El-hameed et al. [52] reported high MDA levels in birds exposed to imidacloprid. On the other hand, supplementation of selenium nano form reduced the MDA level in birds, as stated by Ibrahim et al. [25]. Selenium participates in selenoprotein synthesis, which forms antioxidative enzymes such as glutathione peroxidase [22]. Hence, dietary selenium can relieve oxidative stress from imidacloprid toxicity by enhancing the antioxidative capacity and reducing the MDA level.

## Conclusion

Toxicity with imidacloprid is a severe risk impacting broilers’ health and productivity, thereby interrupting the food chain supply. In this study, dietary selenium nanoparticles relieved the adverse impacts of imidacloprid toxicity by improving feed utilization and growth performance. Furthermore, healthy blood hemato-biochemical features resulted from feeding with selenium nano form. Imidacloprid-induced lipid peroxidation in birds while dietary selenium nano form mediated the oxidative stress and resulted in satisfactory health status.

## Data Availability

The datasets generated during and/or analyzed during the current study are available from the corresponding author upon reasonable request.
